# Association between admission high-sensitivity cardiac troponin T levels and clinical outcomes in acute intracerebral hemorrhage: a prospective cohort study

**DOI:** 10.1186/s12883-026-04750-7

**Published:** 2026-02-25

**Authors:** Quoc Viet Bui, Anh Tuan Nguyen, Viet Hai Nguyen, Tien Dung Nguyen, Xuan Trung Vuong, Hong Son Trinh, Van Hung Nguyen, Minh Thu Vu, Viet Phuong Dao

**Affiliations:** 1 Hanoi Medical University, Hanoi, Vietnam; 2 Stroke Center, Bach Mai Hospital, Hanoi, Vietnam; 3 Emergency Center, Bach Mai Hospital, Hanoi, Vietnam; 4 VNU-University of Medicine and Pharmacy, Hanoi, Vietnam; 5 Stroke Unit, Lao Cai General Hospital, Lao Cai, Vietnam; 6 Emergency Department, Duc Giang General Hospital, Hanoi, Vietnam

**Keywords:** Intracerebral hemorrhage, High-sensitivity cardiac troponin T, Stroke-heart syndrome, Mortality prediction, Functional outcome prognosis

## Abstract

**Background:**

Intracerebral hemorrhage remains one of the most devastating forms of stroke, associated with high mortality and disability. Cardiac injury following ICH, mediated through the brain-heart axis, may serve as a marker of disease severity. High-sensitivity cardiac troponin T, which rises rapidly after myocardial injury, has potential prognostic value in stroke-heart syndrome. The objective of this study was to determine whether elevated high-sensitivity cardiac troponin T levels at hospital admission is associated with early mortality, overall mortality, and poor functional outcome at 90 days in patients with acute intracerebral hemorrhage.

**Methods:**

This is a prospective cohort study, which enrolled 256 patients with acute ICH admitted within 24 h of onset to the Stroke Center, Bach Mai Hospital, from February 2025 to June 2025. Baseline characteristics, hematoma features, comorbidities, and high-sensitivity cardiac troponin T levels were collected immediately upon admission. Clinical outcomes included early mortality (< seven days), overall mortality, and poor functional outcome (modified Rankin Scale score 4–6) were evaluated. Multivariate regression identified independent associations.

**Results:**

Elevated high-sensitivity cardiac troponin T levels was observed in 88 patients (34.4%). These patients had significantly lower Glasgow Coma Scale scores and larger hematoma volumes. Poor functional outcome occurred in 80.7% vs., 53.6% (p-value < 0.001), overall mortality in 65.9% vs., 33.9% (p-value < 0.001), and early mortality in 54.5% vs. 22.6% (p-value < 0.001) when comparing elevated and non-elevated high-sensitivity cardiac troponin T patients, respectively. After multivariable adjustment, elevated high-sensitivity cardiac troponin T (hs-cTnT) was independently associated with poor functional outcome (OR 2.41; 95% CI 1.06–5.49) and early mortality (OR 2.85; 95% CI 1.10–7.42). An association with overall mortality was also observed (OR 2.27; 95% CI 0.97–5.27), although this did not reach statistical significance. In time-to-event analyses, patients with elevated hs-cTnT experienced a significantly shorter restricted mean survival time compared with those without hs-cTnT elevation (13.9 vs. 22.4 days), corresponding to a mean survival difference of approximately 8.5 days (95% CI -11.2 to -5.8; *p* < 0.001).

**Conclusion:**

Admission elevated high-sensitivity cardiac troponin T was associated with adverse outcomes in acute primary intracerebral hemorrhage and showed a notable association with early mortality.

**Supplementary Information:**

The online version contains supplementary material available at 10.1186/s12883-026-04750-7.

## Background

Intracerebral hemorrhage (ICH) accounts for only 10–15% of all strokes but is associated with the highest rates of mortality and long-term disability. Up to 40–50% of affected patients die within the first year after onset, and early neurological deterioration is common [1–3]. Identifying high-risk patients during the early phase is essential to guide monitoring intensity, resourse allocation, and treatment strategies.

Cardiac complications are increasingly recognized after ICH, ranging from troponin elevation, arrhythmias, ECG abnormalities, and Takotsubo syndrome, collectively described as stroke-heart syndrome [4–6]. Cardiac enzyme elevation is observed in approximately one out of five patients with ICH and has been increasingly recognized as a marker of systemic stress. The underlying mechanisms are not fully understood but are believed to involve an imbalance between myocardial oxygen supply and demand, accompanied by excessive sympathetic activation secondary to severe brain injury [7]. This hyperadrenergic state and massive catecholamine release can damage cardiomyocytes through calcium overload, mitochondrial dysfunction, and contraction-band necrosis [8]. In some cases, myocardial injury develops on a background of pre-existing coronary artery disease. Regardless of the mechanism, such cardiac alterations are associated with poor outcomes [9].

Several studies have demonstrated that dynamic changes in cardiac troponin levels in ICH patients predict mortality and poor functional outcomes, including both troponin kinetics and peak concentrations [10, 11]. High-sensitivity cardiac troponin T (hs-cTnT) can be measured immediately upon hospital admission and exhibits early dynamic changes-within the first hour after symptom onset-making it a highly sensitive biomarker for detecting even transient ischemia without overt myocardial necrosis [12, 13]. Hs-cTnT at admission appears to be associated with poor outcomes in patients with intracerebral hemorrhage, although current evidence is still limited.

In Vietnam, patients with acute intracerebral hemorrhage are frequently admitted with severe neurological impairment, and access to comprehensive cardiac evaluation, including serial troponin testing or advanced imaging, may be constrained by resource availability and clinical instability. In this context, the ability of a single, readily available biomarker measured at admission to provide early prognostic information may have particular clinical relevance.

Therefore, this prospective cohort study aimed to evaluate the association between admission hs-cTnT levels and early mortality, overall mortality, and 90-day functional outcome in patients with acute primary intracerebral hemorrhage treated at a tertiary stroke center in Vietnam, with the goal of validating its prognostic utility in a real-world, resource-limited setting.

## Methods

### Aim

To evaluate the prognostic value of hs-cTnT measured at admission in predicting 90-day mortality and poor functional outcome (modified Rankin Scale score 4–6) at in patients with acute ICH.

### Study design

A prospective observational cohort study was conducted at the Stroke Center of Bach Mai Hospital, one of the largest tertiary stroke centers in Vietnam.

### Study population

All patients aged ≥ 16 years who were admitted to the Stroke Center of Bach Mai Hospital within 24 h of symptom onset (or last known well) between February and May 2025 were screened for enrollment. The diagnosis of acute intracerebral hemorrhage was confirmed by non-contrast brain CT csan. Serum hs-cTnT testing was performed immediately upon hospital admission for all eligible patients. Patients were excluded if they had secondary intracerebral hemorrhage due to trauma, vascular malformation, or coagulopathy. Patients with high-risk intracerebral hemorrhage associated with cerebral vascular malformations who did not undergo CT angiography were excluded, primarily due to clinical instability or critical illness precluding the procedure (Supplement Table 1).

### Sample size estimation

**Fig. 1:**
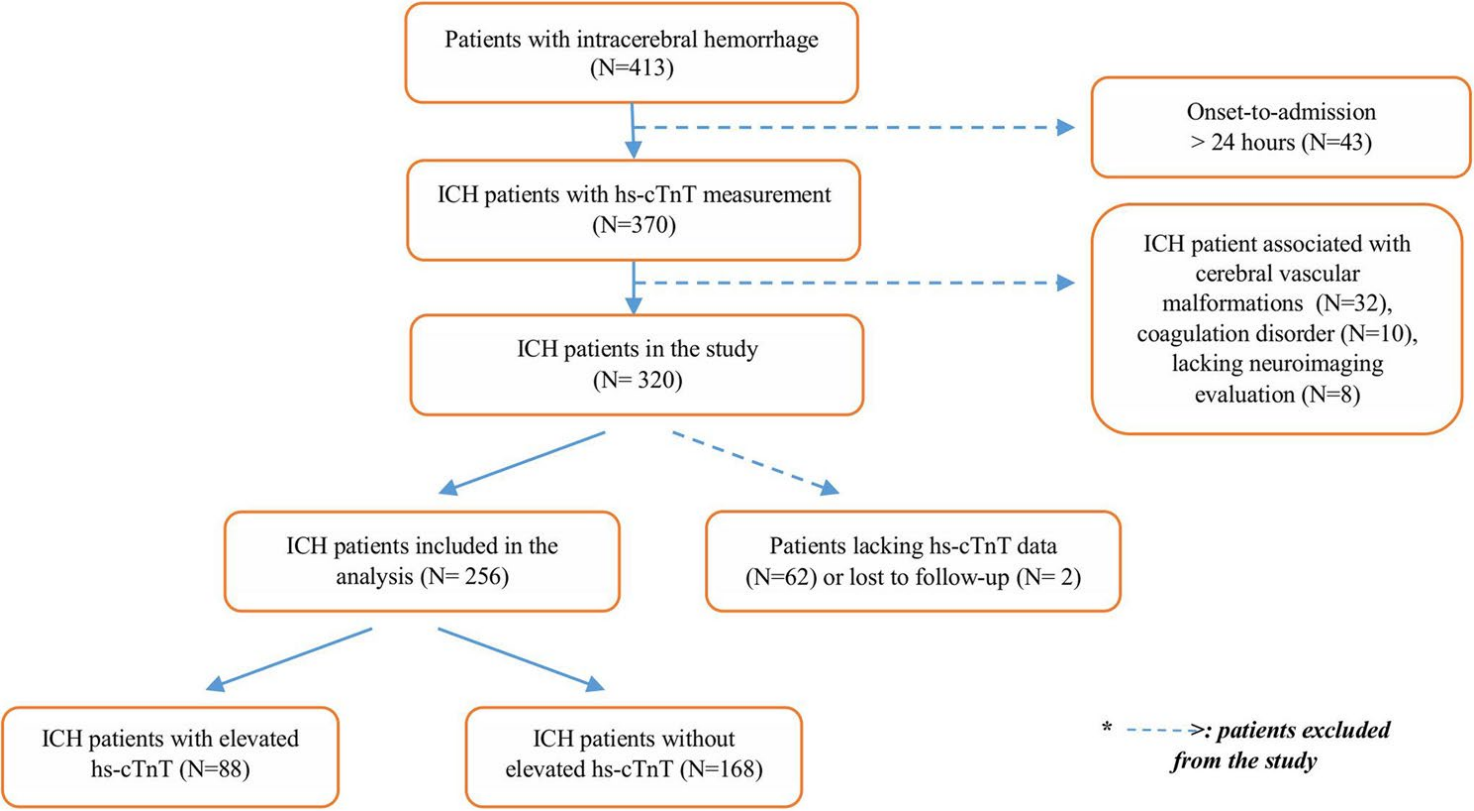
Flowchart of patient selection

The sample size was calculated using the following formula to compare two proportions:

$\:{n}_{1}=\:\frac{{[{Z}_{1-\frac{\alpha\:}{2}}\sqrt{\stackrel{-}{p}\left(1-\:\stackrel{-}{p}\right)\left(1+\:\frac{1}{k}\right)}+\:{Z}_{1-\:\beta\:}\sqrt{{p}_{1}\left(1-\:{p}_{1}\right)+\:\frac{{p}_{2}(1-{p}_{2})}{k}}]}^{2}}{{({p}_{1}-\:{p}_{2})}^{2}}$, where n2 = k × n1, The significance level was set at α = 0.05, and statistical power was defined as 1 − β = 0.80. An allocation ratio of k = 2 was applied between the two groups. The anticipated difference in proportions was assumed to be $\:{p}_{1}-\:{p}_{2}=0.185$. Based on these assumptions, a total sample size of 256 participants was considered sufficient to achieve the desired statistical power (Fig. 1).

### Study process

All patients received standardized medical management according to the 2024 clinical guideline of the Vietnamese Ministry of Health, which is closely aligned with the 2022 American Heart Association/American Stroke Association (AHA/ASA) guideline and the 2025 European Stroke Organisation guideline for spontaneous intracerebral hemorrhage [14, 15]. Invasive diagnostic procedures for suspected acute coronary syndrome were generally avoided, as the use of antithrombotic therapy required for these procedures carries a bleeding risk in the context of acute ICH.

A structured care bundle formed the core of the management strategy. Its main components included intensive blood pressure reduction targeting a systolic range of 130–150 mmHg, optimally below 140 mmHg; correction of coagulopathy when present; and strict control of blood glucose and body temperature. This bundle was applied for at least 24 h and could be extended up to seven days, or until the patient was discharged or treatment was completed [15–17]. Patients with large hematomas underwent neurosurgical evacuation when indicated. Clinical outcomes were evaluated at 90 days using the modified Rankin Scale (mRS) [18], through either in-person evaluation or telephone follow-up interviews. Poor functional outcome was defined as mRS score of 4–6, and early mortality was defined as death within seven days of onset [19]. Serum hs-cTnT was measured immediately upon hospital admission, typically within 30 min of arrival, using the Cobas 8000 analyzer. The assay’s 99th-percentile upper reference limit in healthy individuals is 14 ng/L. Based on this established threshold, hs-cTnT elevation was subsequently defined using kidney function-specific cut-off values to account for the influence of renal impairment. Patients were classified as having elevated hs-cTnT if concentrations exceeded 14, 18, or 48 ng/L among those with an estimated glomerular filtration rate (eGFR) of > 60, 30–60, or < 30 mL/min/1.73 m², respectively. This approach yielded a single binary variable (elevated vs. non-elevated hs-cTnT), which was used consistently in all regression analyses [6, 20–22].

### Statistical analysis

Continuous variables were reported as mean ± standard deviation (SD) for normally distributed data and as median with interquartile range (IQR) for skewed data. Categorical variables were expressed as frequencies and percentages. Missing data were not imputed as patients with incomplete data were excluded from the analysis.

For univariate analysis, patients with and without acute myocardial injury were compared using the independent samples *t*-test for normally distributed continuous variables, the Mann-Whitney *U* test for non-normally distributed or ordinal variables, and the χ² test for categorical variables.

Multivariate logistics regression analysis was performed to explore the independent associations between acute myocardial injury (presence vs. absence) and clinical outcomes, including overall mortality and poor functional outcome; Multivariate Cox regression was used to assess the association with mortality over time. Given the acute nature of ICH, early mortality, defined as death within seven days of onset, was analyzed as a separate outcome. Survival analysis was initially performed using Cox proportional hazards regression. The proportional hazards assumption was formally assessed using Schoenfeld residuals and log-minus-log survival plots. Because the proportional hazards assumption was violated for hs-cTnT, restricted mean survival time (RMST) analysis was subsequently performed to compare survival between groups without assuming proportional hazards. RMST was calculated up to 30 days after intracerebral hemorrhage onset.

Variables included in the multivariate regression model were elevated hs-cTnT, age, Glasgow Coma Scale (GCS) score [23], hematoma volume, and hematoma location. A *p*-value < 0.05 was considered statistically significant. Statistical analyses were conducted using SPSS version 20.0 (IBM Corp, Armonk, NY, USA).

Continuous variables with more than two groups were compared using one-way analysis of variance (ANOVA) for normally distributed data or the Kruskal-Wallis test for non-parametric and ordinal data. Categorical variables were compared across groups using the χ² test.

In addition, descriptive statistics and comparative outcome analyses were performed between the two subgroups: patients with elevated hs-cTnT and those with non-elevated hs-cTnT.

## Results

A total of 256 patients with primary ICH were enrolled in the study, all of whom underwent hs-cTnT testing upon hospital admission. Of these, 193 patients (75.4%) were male. Elevated hs-cTnT was detected in 88 patients (34.3%). All patients were evaluated within 24 h of symptom onset or the last known well time, with a median time to assessment of 5.0 h. Baseline characteristics of the study cohort are summarized in Table 1, comparing patients with elevated hs-cTnT and those without elevation.

**Table 1:** Baseline characteristics of the study population according to hs-cTnT status

Baseline characteristics	Total cohort (*N* = 256)	Elevated hs-cTnT group (*N* = 88) *n* (%)	Non-elevated hs-cTnT group (*N* = 168) *n* (%)	*p* value
Age (years)mean	61.7 ± 12.7	64.3 ± 13.8	60.4 ± 11.9	0,012
Sex (Male)*n* (%)	193 (75.4)	78 (88.6)	115 (68.5)	0.001
Glasgow Coma Scale (GCS)Median (IQR)	10.0 (6.0–14.0)	8.0 (6–12)	11,5 (7–15)	< 0.001
Hypertension*n* (%)	188 (73.4)	69 (78.4)	119 (71.3)	0.2784
Hypertensive emergency at admission*n* (%)	208 (81.3)	77 (87.5)	131 (82.4)	0.3829
Diabetes mellitus*n* (%)	29 (11.3)	14 (15.9)	15 (8.9)	0.143
Atrial fibrillation*n* (%)	4 (1.6)	3 (3.4)	1 (0.6)	0,233
Renal failure* (eGFR≤60 ml/ph/1,73m^2^) *n* (%)	12 (4.7)	9 (10.2)	3 (1.8)	0.006
History of stroke *n* (%)	35 (13.7)	16 (18.2)	19 (11.3)	0.184
Baseline modifiedRankin Scale (pre-mRS),median [IQR]	0 (0–4)	0 (0–3)	0 (0–4)	0.293
Referred from other hospital *n* (%)	145 (56.6)	53 (60.2)	92 (54.8)	0.481
Hematoma volume (ml)	22.0 (8.4–52.1)	24.7 (9.2–66.5)	17.7 (7.3–42.0)	0.018
Time to hospital admission (hour)	5.0 (3.0–7.8)	5.0 (4.0–8.0)	4.5 (3.0–7.5.0.5)	0.154
Surgical evacuation	38 (14.8)	8 (9.1)	30 (17.9)	0.091
Location of hemorrhage *n* (%)				
- Basal ganglia	122 (47.5)	36 (40.9)	86 (51.2)	0,238
- Thalamus/Internal	42 (16.3)	19 (21.6)	23 (13.7)	
- Cortical/Lobar	37 (14.4)	14 (15.9)	23 (13.7)	
- Cerebellum	22 (8.6)	5 (5.7)	17 (10.1)	
- Brainstem	32 (12.5)	14 (15.9)	18 (10.7)	

* *eGFR* Estimated Glomerular Filtration Rate by CKD-EPI equation

**Fig. 2:**
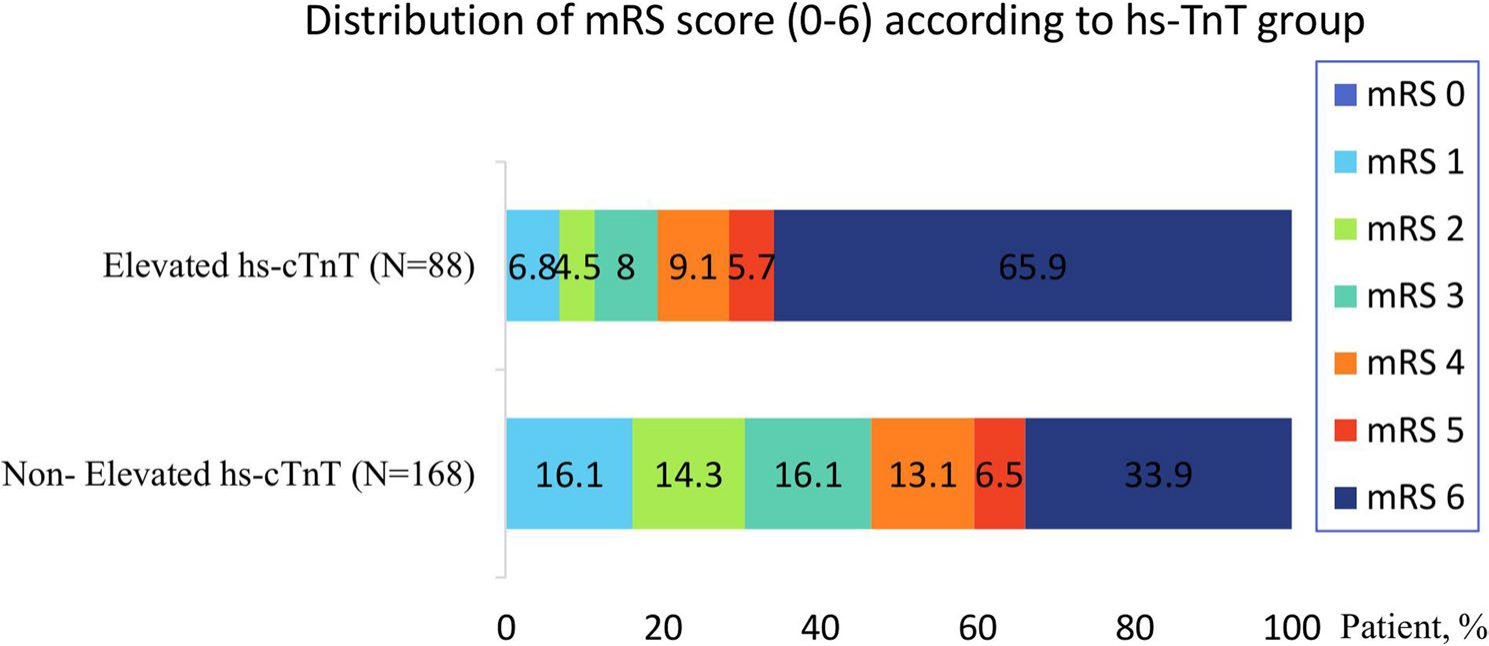
Horizontal stacked bar chart showing the distribution of modified Rankin Scale scores (0–6) according to hs-Troponin T status. Each bar represents the proportion of patients across mRS categories (0–6) for the elevated and non-elevated hs-cTnT groups

The overall clinical outcomes of the study population were evaluated using the mRS, ranging from 0 to 6. The distribution of mRS scores according to hs-cTnT status is illustrated in Fig. 2, presented as a horizontal stacked bar chart. A high mortality rate was observed in both groups, but it was significantly higher in patients with elevated hs-cTnT (65.9%) compared with those without troponin elevation (33.9% - Fig. 2).

Clinical outcomes at 90 days, based on the mRS and early mortality, are summarized in Table 2. Patients with elevated hs-cTnT had significantly worse functional outcomes and higher mortality compared with those without troponin elevation. Specifically, the proportion of patients with poor functional outcome (mRS 4–6) was markedly higher in the elevated hs-cTnT group (80.7% vs. 53.6%, p-value < 0,001), and the overall mortality rate was also significantly increased (65.9% vs. 33.9%, p-value < 0,001).

**Table 2:** Distribution of clinical outcomes according to hs-cTnT status

	Total cohort (*n* = 256) *n* (%)	Elevated hs-cTnT (*n* = 88) *n* (%)	Non-elevated hs-cTnT (*n* = 168) *n* (%)	*p*-value (Chi-square test)
Poor functional outcome (mRS 4–6) *n* (%)	161 (62.9%)	71 (80.7%)	90 (53.6%)	< 0,001
Overall mortality n (%)	115 (44.9%)	58 (65.9%)	57 (33.9%)	< 0.001
Restricted mean survival time (days)		13.9	22.4	< 0.001
Early mortality (< 7 days) n (%)	86(33.5%)	48 (54.5%)	38 (22.6%)	< 0.001

Clinical outcomes at 90 days, including functional outcome assessed by the mRS and mortality, are summarized in Table 2. Patients with elevated hs-cTnT had significantly worse outcomes compared with those without troponin elevation. Specifically, the proportion of patients with poor functional outcome (mRS 4–6) was markedly higher in the elevated hs-cTnT group (80.7% vs. 53.6%, *p* < 0.001), and overall mortality was also significantly increased (65.9% vs. 33.9%, *p* < 0.001).

**Fig. 3:**
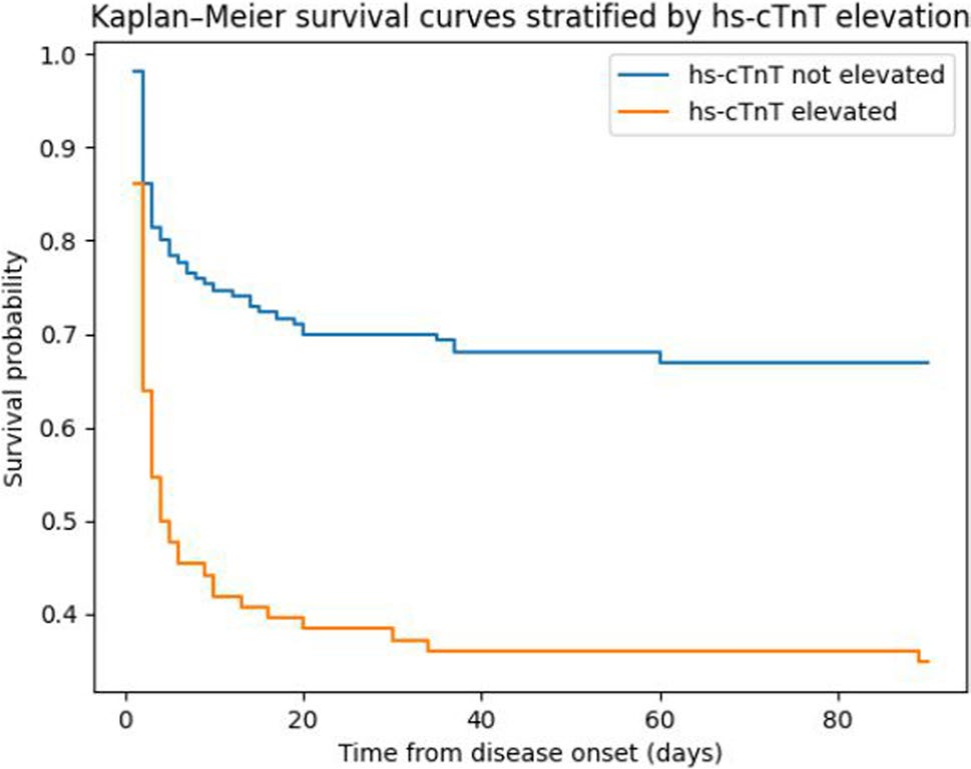
Kaplan–Meier survival curves, stratified according to hs-cTnT status

Formal testing using Schoenfeld residuals demonstrated a violation of the proportional hazards assumption for hs-cTnT (*p* < 0.001), indicating a time-dependent effect. Therefore, Cox proportional hazards regression was not considered appropriate for primary inference, and restricted mean survival time (RMST) analysis was used as the primary approach to evaluate the association between hs-cTnT and mortality. As shown in Table 2, at 30 days, patients with elevated hs-cTnT (> 14 ng/L) had a significantly shorter restricted mean survival time than those without hs-cTnT elevation (13.9 vs. 22.4 days), corresponding to a mean survival difference of approximately 8.5 days (95% CI, − 11.2 to − 5.8; *p* < 0.0001). These findings were further illustrated by Kaplan–Meier survival curves (Fig. 3). Patients with elevated hs-cTnT exhibited a higher cumulative risk of death during follow-up compared with those without troponin elevation.

Table 3 presents the results of the univariable and multivariable logistic regression analyses evaluating factors associated with poor functional outcome (mRS 4–6), overall mortality, and early mortality. After adjustment for relevant clinical and radiological covariates, elevated hs-cTnT remained independently associated with all adverse clinical outcomes. Male sex and brainstem hemorrhage were also associated with increased mortality, whereas age and other hematoma locations were not significantly associated with outcomes.

**Table 3:** Univariate and multivariate logistic regression analysis of factors affecting treatment outcomes

	Poor functional outcome (mRS 4–6) ^*^		Overall mortality ^*^		Early mortality (< seven days)^*^		
	OR	*p* (CI 95%)	OR	*p* (CI 95%)		OR	*p* (CI 95%)
Univariable regression							
GCS (score)	0.64 (0.58–0.70)	< 0.001	0.65 (0.59–0.72)	< 0.001		0.56 (0.48–0.65)	< 0.001
Age (year)	1.01 (0.99–1.03)	0.551	1.00 (0.99–1.02)	0.689		1.00 (0.98–1.02)	0.949
Hematoma volume (ml)	1.04 (1.03–1.06)	< 0.001	1.02 (1.01–1.03)	< 0.001		1.02 (1.01–1.02)	< 0.001
Elevated hs-cTnT	3.62 (1.97–6.66)	< 0.001	3.77 (2.19–6.49)	< 0.001		4.11 (2.36–7.14)	< 0.001
Gender (Male)	1.64 (0.92–2.93)	0.093	2.08 (1.14–3.80)	0.017		1.86 (0.97–3.57)	0.061
eGFR (ml/ph/1,73m^2^)	0.99 (0.98–1.00.98.00)	0.021	0.99 (0.97–1.00.97.00)	0.007		0.98 (0.97–1.00.97.00)	0.006
Hematoma location ^#^							
Brainstem	2.91 (1.12–7.59)	0.029	5.59 (2.19–14.27)	< 0.001		2.32 (1.05–5.12)	0.037
Cerebellum	0.97 (0.38–2.44)	0.948	3.03 (1.11–8.27)	0.031		1.42 (0.56–3.60)	0.461
Cortical/Lobar	1.81 (0.81–4.08)	0.151	0.28 (0.10–0.78)	0.014		0.87 (0.39–1.93)	0.727
Thalamus/Internal	0.74 (0.36–1.50)	0.399	1.52 (0.64–3.61)	0.340		0.56 (0.24–1.28)	0.169
Multivariate regression ^$^							
Elevated hs-cTnT	2.41 (1.06–5.49)	0.036	2.27 (0.97–5.27)	0.058		2.85 (1.10–7.42)	0.031

*OR, *odds ratio,* CI *confidence interval,* hs-cTnT *high-sensitivity cardiac troponin T

* Logistic regression analysis

#Compared with the reference group - deep intracerebral hemorrhage involving the basal ganglia (deep location)

$ Variables included in the model: male sex, Glasgow Coma Scale score, hematoma volume, hemorrhage location, eGFR and hs-cTnT elevation

## Discussion

In this study, hs-cTnT measured at admission was associated with poor functional outcome and early mortality in patients with acute ICH. The lack of statistical significance for overall mortality may be related to the modest sample size and limited number of events, although the magnitude of the effect suggests potential clinical relevance. After adjustment for established prognostic factors, including age, GCS score, hematoma volume, renal impairment, and hemorrhage location, elevated hs-cTnT remained consistently associated with adverse clinical outcomes across all endpoints. These findings suggest that hs-cTnT elevation may reflect disease severity and is closely linked to early clinical deterioration and subsequent disability in patients with ICH.

Our findings are consistent with prior studies demonstrating an association between troponin elevation and mortality in ICH. Yangchun He et al. [11] reported that increased troponin I was associated with a more than threefold increase in in-hospital mortality, while Ulger et al. [24] identified a substantial increase in mortality risk among patients with troponin I concentrations greater than 26 ng/L [11, 24]. Retrospective analyses of the FAST trial by He [11], Ulger [24], and Rosso [10] further highlighted the prognostic relevance of troponin dynamics in patients with ICH [10, 11, 24]. The absence of a significant association between admission troponin I and outcomes among surgically treated ICH patients reported by Akin et al. (2025) likely reflects differences in patient populations, particularly the predominance of surgically managed cases, as well as variation in the timing and frequency of biomarker assessment [25]. In our cohort, less than 15% of patients underwent hematoma evacuation, limiting the power to explore surgical subgroups.

The exact mechanism underlying troponin elevation in ICH remains unclear [26]. Experimental and clinical evidence suggests that acute neurological injury can trigger profound sympathetic overactivation, catecholamine surge, and microvascular dysfunction, resulting in myocardial injury that is predominantly neurogenic rather than ischemic in origin. In this context, troponin elevation may serve as a biomarker of global physiological stress and the severity of brain–heart interaction [27, 28]. Accumulating evidence also indicates that the anatomical location of brain injury plays a critical role in this process. Hemorrhagic involvement of autonomic regulatory regions, particularly the insular cortex, has been associated with exaggerated sympathetic discharge and catecholamine release. Damage to the insula—especially in the right hemisphere—may disrupt central autonomic balance, leading to neurogenic stress cardiomyopathy with Takotsubo-like features, characterized by myocardial stunning and troponin elevation in the absence of primary coronary ischemia [27]. Elevated hs-cTnT was significantly associated with lower GCS scores and larger hematoma volumes, supporting the hypothesis that hs-cTnT serves as a marker of global injury severity. Although the present study was not designed to directly evaluate lesion-specific autonomic involvement, these mechanisms provide a biologically plausible framework to interpret the observed association between hs-cTnT elevation and adverse outcomes.

Traditional predictors incorporated in the ICH score, including GCS, hematoma volume, hemorrhage location, and age-remain independent prognostic factors for mortality [29]. However, our findings indicate that hs-cTnT may provide additional prognostic information for both mortality and poor functional outcome. As a readily available biomarker, hs-cTnT measurement at admission could assist clinicians in early risk stratification and prognostication in patients with acute ICH.

Our cohort presented with more severe clinical features compared with those reported in other studies, with a median GCS score of 10 and a median hematoma volume of 22.0 mL. Bach Mai Hospital, the largest tertiary stroke center in northern Vietnam, receives a high proportion of severe referral cases (44.9%), which likely contributed to the high early mortality rate observed in our study (33.5%). This may explain why mortality exceeded that reported by Mai Duy Ton et al [30]. (21% across eight stroke centers) and global estimates from Poon et al. (2014), where one-year survival after ICH was approximately 54% [30, 31]. Despite these differences, the prognostic association of hs-cTnT remained consistent across all analyses, suggesting that its utility may extend to populations with more severe disease.

In the context of a demonstrated violation of the proportional hazards assumption, RMST analysis was selected as the primary approach for evaluating time-to-event outcomes. The significantly shorter RMST observed among patients with elevated hs-cTnT provides a clinically intuitive measure of survival disadvantage during the early phase of acute ICH. Our study has several limitations. Firstly, troponin measurement was performed only once in most patients, largely due to the rapid clinical deterioration of severe cases and the associated laboratory costs. Therefore, the dynamic changes in troponin levels over time could not be fully evaluated, and serial testing was only available in a limited number of patients. Secondly, additional cardiac evaluations, such as echocardiography or coronary angiography, were not routinely performed in patients with elevated troponin levels, which may have led to underdiagnosis of true myocardial infarction requiring intervention. Thirdly, the study population represented relatively severe cases, and mortality rates were high in both the troponin-positive and troponin-negative groups. This reflects the unpredictable nature of the brain-heart interaction, as not all patients with large hematomas necessarily develop cardiac injury. Fourth, a subset of patients with intracerebral hemorrhage was excluded because computed tomography angiography could not be safely performed, most often due to critical clinical instability. The exclusion of these severely ill patients may have influenced the observed mortality rate and, consequently, the overall analytical findings to some extent. Finally, although both cardiac troponin T and I have comparable diagnostic value for myocardial injury, high-sensitivity troponin T may increase in patients with renal impairment or other comorbid conditions, potentially confounding the interpretation of troponin elevation [32, 33]. High-sensitivity troponin I might therefore be a promising marker for future studies.

## Conclusion

Overall, our findings demonstrate that hs-cTnT measured at admission is a biomarker independently associated with poor functional outcome and mortality in patients with acute ICH. The results in this study suggest that incorporating hs-cTnT into early assessment workflows, alongside established predictors such as GCS, age, and hematoma volume, may improve risk stratification, facilitate timely clinical decision-making, and identify patients who may benefit from enhanced monitoring or advanced supportive therapies.

## Supplementary Information

Supplementary Material 1

## Data Availability

The datasets used and/or analysed during the current study are available from the corresponding author on reasonable request.

## Abbreviations

ICH: Intracerebral hemorrhage
hs-cTnT: High-sensitivity cardiac troponin T
AHA: American heart association
ASA: American stroke association
mRS: Modified rankin scale
SD: Standard deviation
IQR: Interquartile range
GCS: Glasgow coma scale
ANOVA: Analysis of variance
