# Supplementary material for: Association between admission high-sensitivity cardiac troponin T levels and clinical outcomes in acute intracerebral hemorrhage: a prospective cohort study
Source: BMC Neurol. 2026 Feb 25;26:177. doi: 10.1186/s12883-026-04750-7 (PMC13001354; doi:10.1186/s12883-026-04750-7)
Supplement: Supplementary file 1 — Supplementary Material 1 [file 12883_2026_4750_MOESM1_ESM.pdf]

Supplementary Table 1: Comparison of baseline characteristics between patients excluded due to lacking admission high-sensitivity cardiac troponin T (hs-cTnT) or loss to follow-up and the enrolled patients

**Baseline characteristics**	**Total cohort (N= 256)**	**Exclusion (N=64)**	**p value**
Age (years)<br>mean	61.7 ± 12.7	62.4 ± 10.4	0,102
Sex (Male)<br>n (%)	193 (75.4)	45(70.3)	0.126
Glasgow Coma Scale (GCS)<br>Median (IQR)	10.0 (6.0 – 14.0)	10 (6.0-14.0)	
Baseline modified Rankin Scale (pre-mRS), median [IQR]	0 (0–4)	0 (0–4)	0.324
Referred from other hospital<br>n (%)	145 (56.6)	38(59.3)	0.481
Hematoma volume<br>(ml)	22.0 (8.4 – 52.1)	21.2 (7.8-53.3)	0.318
Time to hospital admission (hour)	5.0 (3.0 – 7.8)	5.0(4.0-8.0)	0.214

As shown in Supplementary Table 1, there were no statistically significant differences between the excluded and enrolled patients; therefore, exclusion is unlikely to have materially affected the mortality outcomes of the study population.
